# Case Report: Twists and turns: a case of cervical mesonephric adenocarcinoma on contrast-enhanced ultrasound

**DOI:** 10.3389/fonc.2026.1726429

**Published:** 2026-02-06

**Authors:** Qiuyun Huang, Yunhao Luo, Jia Xu, Kewen Ding, Fangqin Liu, Lang Qiao

**Affiliations:** 1 Function Department of Sichuan Integrative Medicine Hospital, Chengdu, China; 2 Chengdu University of Traditional Chinese Medicine, Chengdu, China

**Keywords:** cervical adenocarcinoma, CEUS, contrast-enhanced ultrasound, mesonephric adenocarcinoma, SonoVue

## Abstract

Cervical mesonephric adenocarcinoma is an extremely rare malignant tumor that originates from the mesonephric duct remnants on the lateral wall of the cervix and is a unique subtype of cervical epithelial tumors. Due to its rarity and non-specific clinical manifestations, early diagnosis is challenging. We report a case of 61-year-old female patient who presented with vaginal bleeding lasting over one month. Following conventional transvaginal ultrasound, contrast-enhanced ultrasound (CEUS), and magnetic resonance imaging (MRI) examination, MRI preliminary diagnosis suggested inflammatory lesions, while CEUS indicated malignant tumors. Finally, the patient underwent surgical treatment, and the pathological diagnosis was mesonephric adenocarcinoma of the cervix. Previous studies have shown that cervical mesonephric adenocarcinoma, often appears atypical on two-dimensional and color Doppler ultrasound. CEUS offers significant advantages in displaying the microvascular architecture of tumors and can provide more detailed and abundant perfusion information, thereby helping to improve the accuracy of diagnosis.

## 1 Introduction

Cervical mesonephric adenocarcinoma is a rare tumor arising from remnants of the mesonephric duct located on the lateral wall of the cervix, which is a special type of cervical epithelial tumor (1). It accounts for less than 1% of all cervical adenocarcinomas (2), with only a limited number of cases reported in the literature. Its biological behavior remains unclear, making early diagnosis of great significance. At present, there is still a lack of unified criteria for characteristic imaging diagnosis. CEUS has the advantages of real-time, dynamic and radiation-free, enables clear visualization of microvascular perfusion, thus contributing to diagnosis.

## 2 Case presentation

### 2.1 Medical history

**Figure 1:**
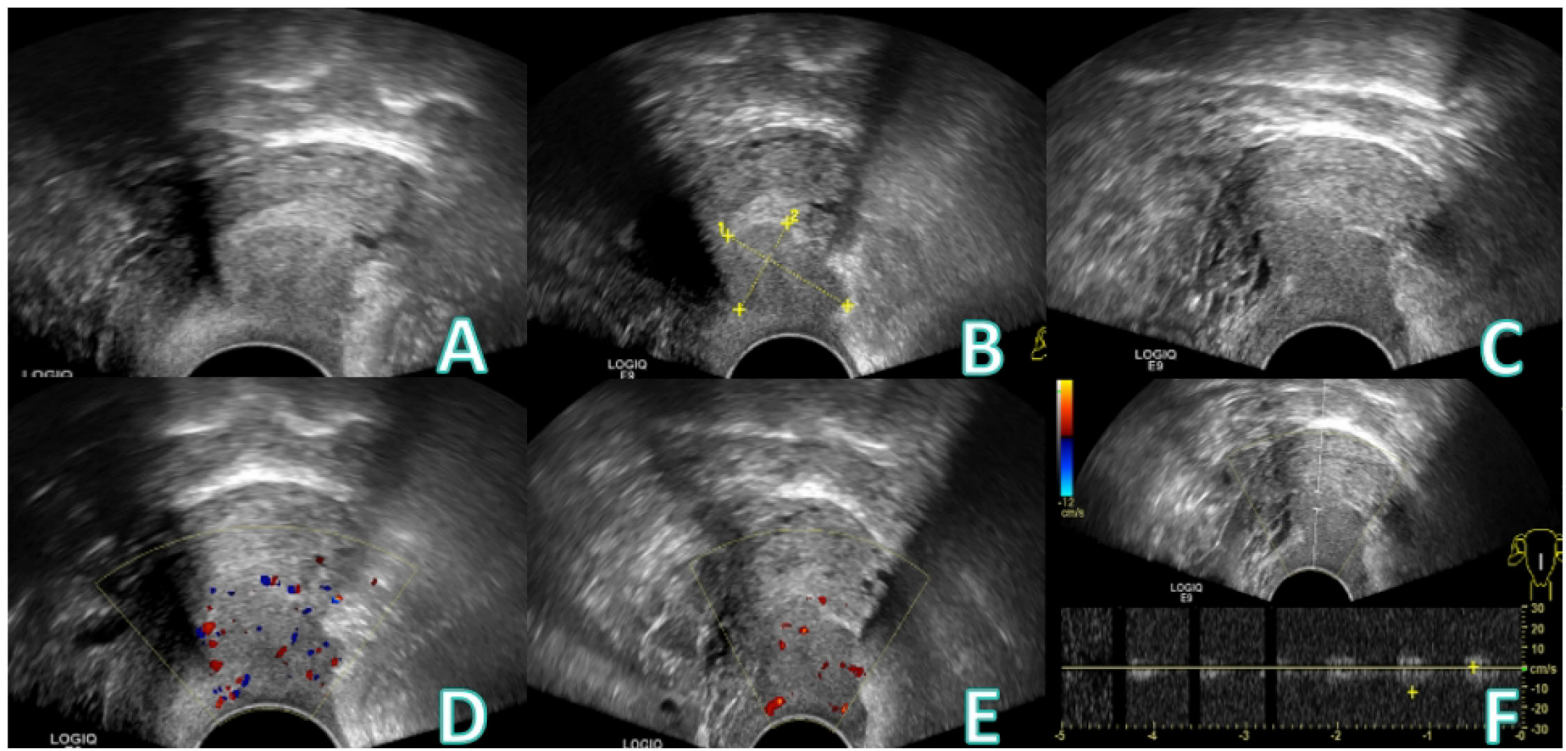
Conventional transvaginal ultrasound images: **(A-C)** A hypoechoic nodule, approximately 2.5×1.8 cm in size, is seen posterior to the uterus and poorly demarcated from it. **(D)** A few punctate blood flow signals can be seen in color Doppler. **(E)** Punctate blood flow signals are visible on spectral Doppler. **(F)** Arterial blood flow signal was detected.

**Figure 2:**
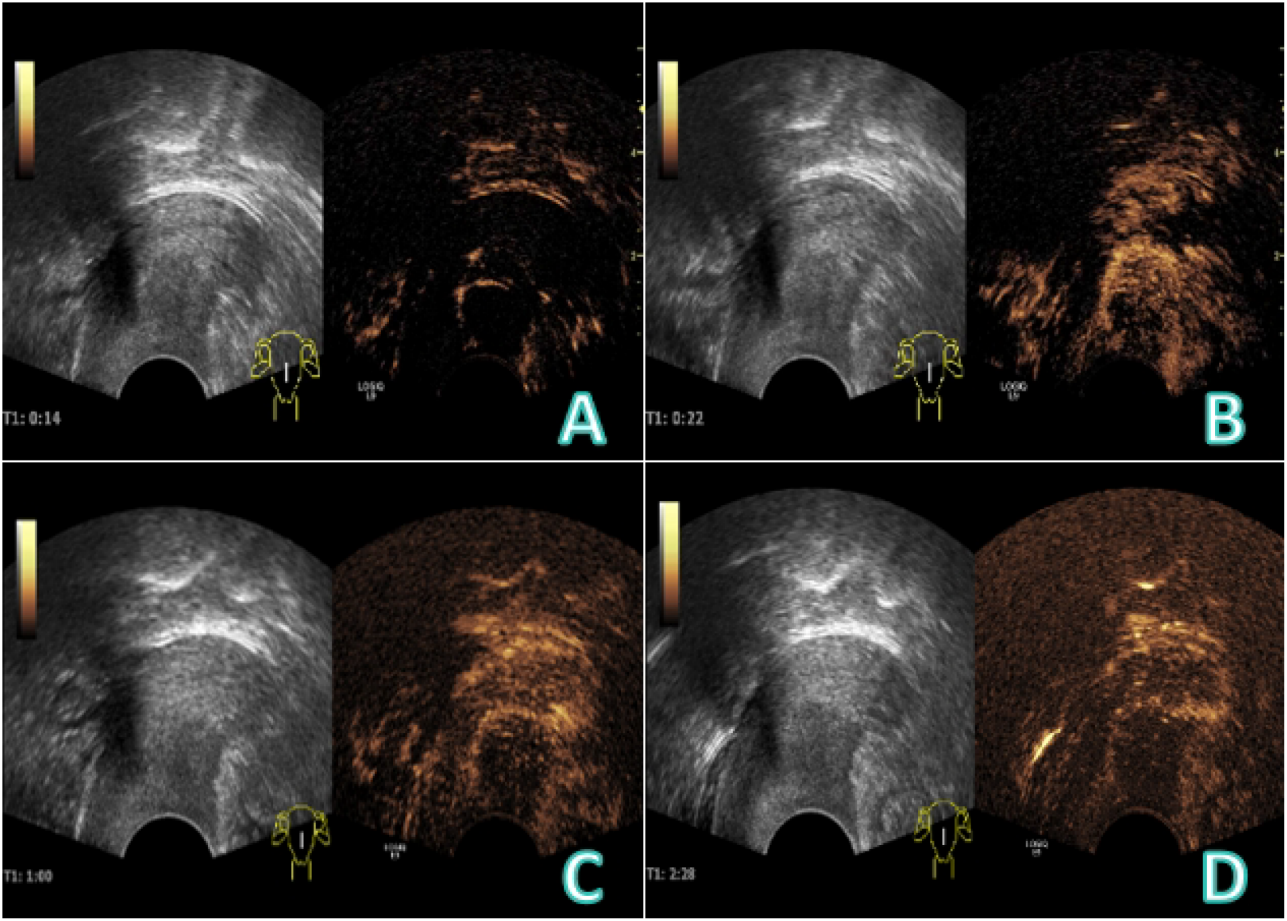
CEUS image: **(A)** At 14 s, the nodule showed semi−circular enhancement, and internal transverse thick feeding vessels connecting with the cervix were visible, demonstrating rapid hyperenhancement. **(B)** At 22 s, the nodule was partially filled from the periphery to the center, with overall heterogeneous hyperenhancement. **(C, D)** In the late enhancement phase, the nodule showed heterogeneous hypoenhancement, with persistent peripheral ring enhancement.

**Figure 3:**
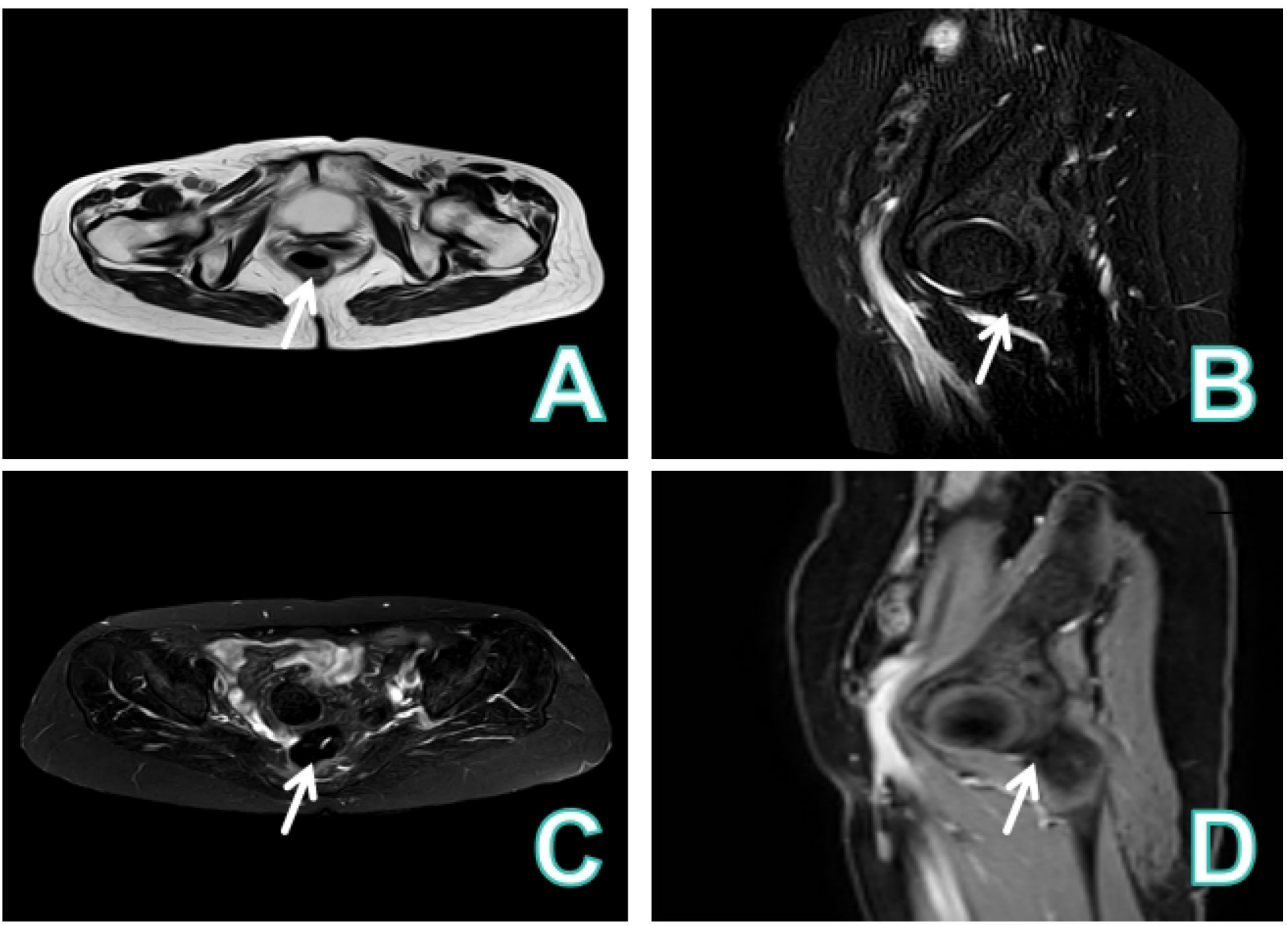
Contrast-enhanced MRI image. **(A)** The vaginal wall was thickened and enhanced, and the posterior fornix showed a mass with long T1 and slightly long T2 signals. **(B-D)** The lesion was close to the posterior uterine wall and had a clear boundary with the rectum. Diffusion of the lesion was restricted, and enhanced scan revealed heterogeneous enhancement with obvious peripheral enhancement, making abscess a possible consideration.

A 61−year−old female presented with vaginal bleeding for over one month. The patient reported experiencing scant vaginal bleeding without an obvious precipitating factor for more than one month, accompanied by mild dizziness and fatigue. Her menstrual history was menarche at age 12 and menopause at age 48,with a gravida and para status of G1P1.She had undergone vaginal repair surgery in 2008.Specialized physical examination revealed vulva of parous type, a moderate amount of bloody discharge within the vagina, and a defect with bleeding on the posterior vaginal wall. Laboratory investigations indicated anemia (RBC 3.13×10^12/L), hypoalbuminemia (Alb 30 g/L), and elevated inflammatory markers(CRP 109 mg/L).Tumor markers were not assessed. The patient underwent conventional transvaginal ultrasound examination (**Figure 1**), CEUS, intravenously injected Sonovue 2.0ml via the elbow vein, (**Figure 2**), and contrast-enhanced MRI (**Figure 3**).

### 2.2 The process of pathological diagnosis and surgical findings

Due to the discrepancy between CEUS and MRI findings, the patient underwent transvaginal nodule biopsy. The biopsy pathology indicated adenocarcinoma. To identify the primary tumor origin, systemic examination (e.g., chest CT) was performed to exclude metastasis, followed by consideration of a primary vaginal tumor such as mesonephric adenocarcinoma. After completing the staging workup and ruling out distant metastasis, the patient subsequently underwent surgery.

Intraoperative findings: extensive adhesions between the greater omentum and the anterior abdominal wall, as well as dense membranous adhesions involving part of the intestine and the bilateral adnexa/pelvic sidewalls. A firm area was noted in the posterior vaginal fornix, with a centrally located ulcerative lesion approximately 1 cm in diameter that involved the full thickness of the vaginal wall.

A discrepancy existed between the intraoperative frozen section and the final postoperative pathological diagnoses:

Intraoperative Frozen Section Diagnosis: (Vagina) Mesonephric adenocarcinoma. Combined with the clinical findings, the staging was considered FIGO stage IIA (tumor invading the vaginal wall but not the parametrial tissue).

**Figure 4:**
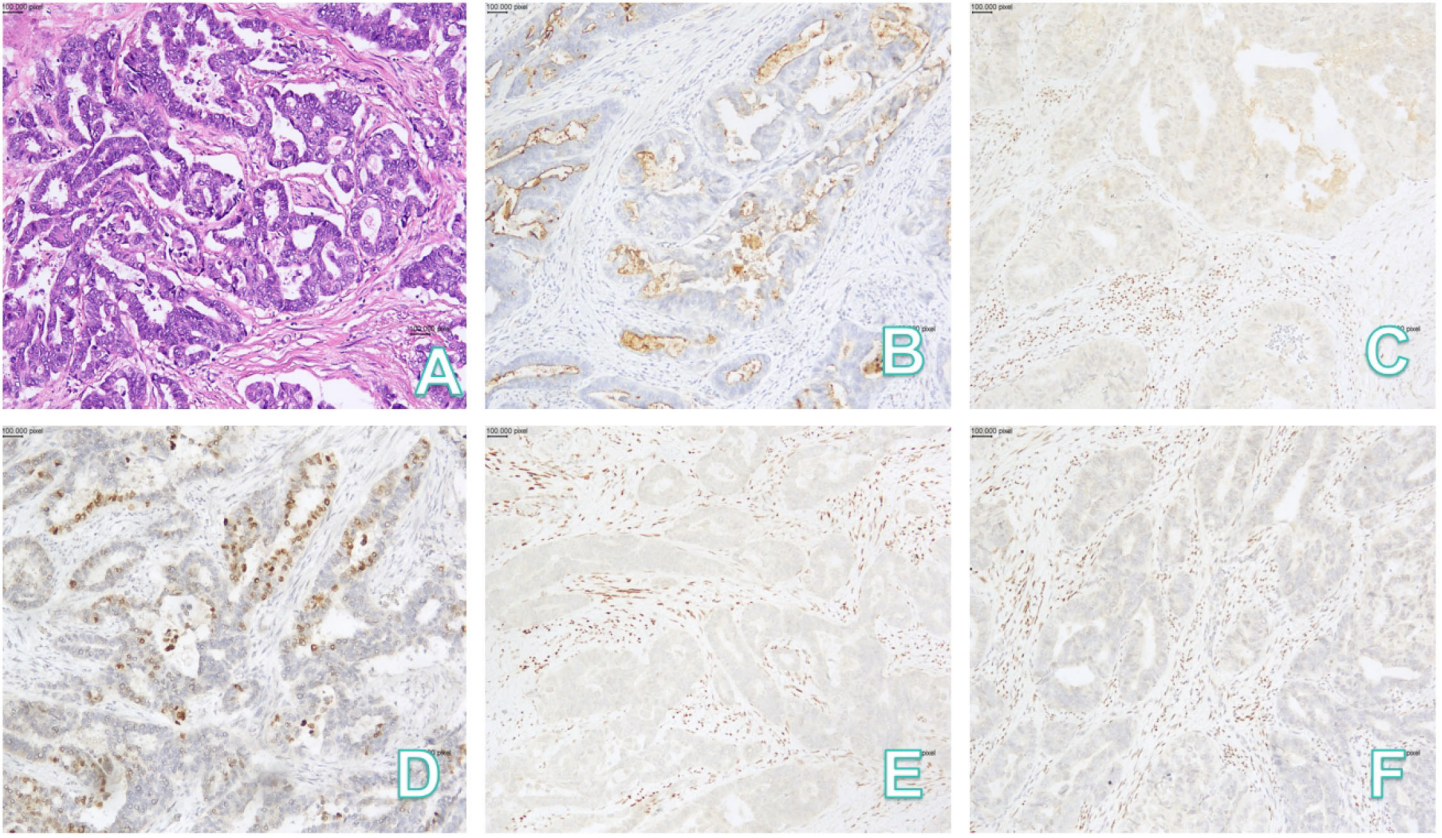
Pathology: **(A)** HE(x100); **(B)** CD10(x100); **(C)** ER(x100); **(D)** GATA-3(x100); **(E)** PR(x100); **(F)** WT-1(x100). HE, hematoxylin-eosin.

Postoperative Paraffin Section Diagnosis (Final):(Cervix) Mesonephric duct adenocarcinoma. Histologic examination revealed that the tumor arose from the deep cervical stroma, with full-thickness stromal invasion. Lymphovascular space invasion and perineural invasion were present. The tumor involved the vaginal fornix stroma, the cervical-uterine junction, and the deep muscle wall of the lower uterine segment (invasion depth >1/2 of the myometrial thickness), extending to the surgical margin of the lateral pelvic wall. Tumor thrombus was identified within parametrial vessels. The vaginal surgical margin, parametrial soft tissue, bilateral adnexa, and all examined (right pelvic) lymph nodes (0/14) were free of tumor. The pathology are shown in **Figure 4**. Immunohistochemical Results: EMA (++), GATA-3 (+), TTF-1 (+), CD10 (+), p53 (wild-type pattern), p16 (patchy +), PAX-8 (+), ER (-), PR (-), WT-1 (-). The Ki-67 proliferation index was approximately 45%.

Based on the final paraffin pathology, the primary tumor site was revised from the vagina to the cervix.

## 3 Discussion

The residual tissue of the mesonephric duct is typically located in the parametrial region (paracervical area), lateral cervical wall, and cervical stroma. Mesonephric adenocarcinoma primarily arises in the cervix (3).It is a rare tumor, accounting for less than 1% of cervical adenocarcinomas (4), usually not associated with HPV infection. Most patients present with abnormal vaginal bleeding, often accompanied by visible cervical lesions, typically occurring in the lateral cervical wall (5, 6), with an average age of onset of 53 years (7). These lesions are often difficult to detect on cytological smears, making histopathological examination the diagnostic gold standard. Its heterogeneous cellular morphology frequently leads to confusion with serous, clear-cell, or endometrioid adenocarcinomas; the most common histological pattern is ductal, while other variants include reticular, solid/tubular, and sex cord-like patterns, with atypical mesonephric hyperplasia commonly found at the tumor periphery or within it. Immunohistochemically, GATA-3 shows nuclear expression in mesonephric lesions but is negative in normal cervical epithelium, endometrial epithelium, and cervical/endometrial adenocarcinomas, aiding differentiation (8–10).; PAX8 is typically strongly positive (11), whereas CK20 and ER are mostly negative, further supporting a mesonephric origin (12).There are no specific treatment protocols; management generally follows guidelines for cervical adenocarcinoma of corresponding clinicopathological stage.

The patient was asymptomatic during a routine check-up one year prior. She presented one year later with irregular vaginal bleeding. Physical examination revealed a bleeding lesion on the posterior vaginal wall. A subsequent conventional transvaginal ultrasound identified a hypoechoic nodule posterior to the uterus, with internal punctate blood flow signals. On pelvic enhanced MRI, the lesion demonstrated slight hypointensity on T1-weighted images and slight hyperintensity on T2-weighted images. Restricted diffusion was noted on DWI. Dynamic contrast-enhanced imaging indicated progressive heterogeneous enhancement, primarily peripheral in distribution. In conjunction with the clinical findings of a ruptured and bleeding posterior vaginal wall, the MRI features were primarily consistent with a chronic abscess formation (13).

CEUS provides critical diagnostic value by enabling real-time dynamic assessment of lesion hemodynamics, thereby clarifying its origin and nature. In this case, the early CEUS phase clearly demonstrated that the large feeding vessels of the mass originated from the cervix, confirming its cervical origin. In the venous phase, the nodule showed heterogeneous hypo-enhancement with persistent peripheral ring-like enhancement, a pattern comparable to that of cervical leiomyoma. Previous studies report that larger leiomyomas often exhibit peripheral enhancement with homogeneous or heterogeneous iso- to mild hyper-enhancement in the early phase, while smaller fibroids frequently enhance synchronously and uniformly due to a less prominent pseudocapsule (14).In the late washout phase, most fibroids clear synchronously with the myometrium, though the peripheral capsule may retain a “vascular ring sign” (15, 16). Importantly, no necrotic or honeycomb-like enhancement was observed post-contrast, arguing against an abscess and supporting a neoplastic lesion. Although the annular enhancement here resembled that of a fibroid, the lesion displayed “fast-in and fast-out”kinetics rather than the typical”slow-in and slow-out”pattern of fibroids. Conversely, cervical carcinoma usually demonstrates early rapid homogeneous or heterogeneous hyperenhancement followed by rapid washout with high peak intensity (15). Although the “fast-in and fast-out”pattern in this case partially overlaps with that of cervical cancer, the absence of early rapid hyperenhancement means it does not fully conform to the classic enhancement profile of cervical carcinoma. As a pure blood-pool imaging modality, CEUS allows real-time evaluation of lesion morphology, microvascular architecture, and tissue perfusion, significantly improving diagnostic accuracy (17).

In summary, the key diagnostic directions highlighted by CEUS are: ① identification of the lesion’s cervical origin based on feeding vessel anatomy; ② exclusion of an abscess due to the lack of honeycomb-like enhancement; and ③ the coexistence of a benign-like ring enhancement pattern with malignant-like rapid washout kinetics. Although the overall presentation is atypical, the “fast-in and fast-out”pattern warrants strong suspicion for malignancy. Therefore, this cervical neoplasm should be considered a rare malignant subtype.

## 4 Conclusions

Although no definitive CEUS features are specific to cervical mesonephric adenocarcinoma, its patterns—such as fast wash−in and wash−out or early clearance—generally align with the common hemodynamic characteristics of malignant tumors. In cases of masses with an unknown primary, CEUS facilitates the real-time, dynamic assessment of microvascular perfusion patterns and can clearly depict the origin and distribution of tumor-feeding vessels. The utility of CEUS lies in its ability to provide rich diagnostic data on enhancement morphology, intensity, and perfusion, which assists in tissue characterization and source identification, consequently enhancing diagnostic precision.

## Data Availability

The original contributions presented in the study are included in the article/aupplementary material. Further inquiries can be directed to the corresponding author.
